# Computer Mouse Movements as an Indicator of Work Stress: Longitudinal Observational Field Study

**DOI:** 10.2196/27121

**Published:** 2021-04-02

**Authors:** Nicolas Banholzer, Stefan Feuerriegel, Elgar Fleisch, Georg Friedrich Bauer, Tobias Kowatsch

**Affiliations:** 1 Department of Management, Technology and Economics ETH Zurich Zurich Switzerland; 2 Centre for Digital Health Interventions Department of Management, Technology and Economics ETH Zurich Zurich Switzerland; 3 Centre for Digital Health Interventions Institute of Technology Management University of St. Gallen Zurich Switzerland; 4 Center for Salutogenesis Epidemiology, Biostatistics and Prevention Institute University of Zurich Zurich Switzerland

**Keywords:** work stress, psychological stress, stress indicator, computer mouse movements, human-computer interactions

## Abstract

**Background:**

Work stress affects individual health and well-being. These negative effects could be mitigated through regular monitoring of employees’ stress. Such monitoring becomes even more important as the digital transformation of the economy implies profound changes in working conditions.

**Objective:**

The goal of this study was to investigate the association between computer mouse movements and work stress in the field.

**Methods:**

We hypothesized that stress is associated with a speed-accuracy trade-off in computer mouse movements. To test this hypothesis, we conducted a longitudinal field study at a large business organization, where computer mouse movements from regular work activities were monitored over 7 weeks; the study included 70 subjects and 1829 observations. A Bayesian regression model was used to estimate whether self-reported acute work stress was associated with a speed-accuracy trade-off in computer mouse movements.

**Results:**

There was a negative association between stress and the two-way interaction term of mouse speed and accuracy (mean −0.32, 95% highest posterior density interval −0.58 to −0.08), which means that stress was associated with a speed-accuracy trade-off. The estimated association was not sensitive to different processing of the data and remained negative after controlling for the demographics, health, and personality traits of subjects.

**Conclusions:**

Self-reported acute stress is associated with computer mouse movements, specifically in the form of a speed-accuracy trade-off. This finding suggests that the regular analysis of computer mouse movements could indicate work stress.

## Introduction

Stress in the workplace is responsible for over 120,000 deaths and US $187 billion in annual health care spending in the United States [1]. To mitigate this burden, work stress must be monitored and managed. The need for workplace stress management increases even further as the digital transformation of the economy implies profound changes in working conditions [2]. At the same time, digital transformation offers opportunities for better stress management. Human-computer interactions with ubiquitous digital devices could be used for real-time monitoring of work-related stress. In particular, it has been shown that the computer mouse responds to changes in muscular activity as a result of stress [3-6]. Thus, previous studies have investigated the association between stress and the use of the computer mouse [7-11], for instance, by analyzing computer mouse movements [8,10,11]. However, the evidence from these studies is, so far, based on lab experiments using artificially designed computer tasks. Hence, it remains unclear whether an association between stress and the use of the computer mouse can also be observed in the field.

For this study, we hypothesized that there is an association between stress and computer mouse movements. Our hypothesized association is based on the theory of neuromotor noise [12-16]. Stress, induced by time pressure or multitasking, leads to higher neuromotor noise [15,16], which is the noise in control signals steering motor movements. Lower signal-to-noise ratios and limited capacity to process information lead to adaptive movement behavior [12]. For instance, if subjects are required to execute fast movements, then neuromotor noise will lead to greater variability in the direction of movement [15]. The reason for this is that high or low execution speeds induce neuromotor noise, which makes it more difficult to hit the intended target of the movement accurately and requires more or fewer corrections, respectively, along the trajectory [13,14]. That is, the accuracy of the movement has to adjust relative to the movement speed.

In short, the previous literature suggests that stress induces neuromotor noise, resulting in a speed-accuracy trade-off in motor movements. This trade-off is particularly documented in rapid aimed movements [13,14]; based on this, we can expect that it also applies to computer mouse movements. We tested our hypothesis with data from a longitudinal observational field study that included 70 subjects and 1829 observations. Thereby, we collected computer mouse movements and self-reported stress levels from employees during their regular office work for 7 weeks. Using a Bayesian regression model, we present findings that support our hypothesis that work stress is characterized by a speed-accuracy trade-off in computer mouse movements.

## Methods

### Study Design

A 7-week longitudinal field study was conducted at a large European technology company. The company’s human resources director asked 496 employees from different service units (ie, accounting, human resources, information technology, marketing, quality management, logistics, and business development) to participate through an email invitation. The invitation described the study’s objective of understanding the association between computer mouse movements and work stress.

Subjects were not offered financial incentives. However, they were invited to a debriefing event at the end of the study, where the aggregated results were presented. Further, their self-reports were made available to them through graphical diagrams so they could monitor their stress levels over the course of the study.

Among all invited employees, 71 subjects decided to participate. They installed our study software by clicking on a link in the invitation. When subjects first opened the study software, a tutorial explained how the software was used to report stress. During the 7-week study period, the study software asked subjects twice a day to report their stress level. The timings were randomly triggered by our software, namely, once between 9 AM and 11 AM and once between 2 PM and 4 PM. Prior to these self-reports, our software recorded all computer mouse movements for 30 minutes. If subjects were not using their computer at that time (eg, due to a meeting), then no data were recorded.

Data about subjects’ computer mouse movements and self-reports were securely transferred to a server at the organization, from which they were gathered by our research team to perform subsequent analyses. At the beginning of the study, subjects were further asked to report their sociodemographics (ie, age, gender, and education), behavioral attributes regarding health and nutrition (ie, sports, nutrition, smoking, and drinking habits), and expression of the big five personality traits as measured by an established inventory [17]. All variables are described in Table 1.

**Table 1:** Variables and descriptions.

Variable		Description
**Target variable**		
	Valence	Self-reported valence on a scale from 1 (low) to 7 (high)
	Arousal	Self-reported arousal on a scale from 1 (low) to 7 (high)
	Stress	Dummy with 1 if valence <4 and arousal >4 (stress), 0 otherwise (no stress)
**Mouse movements**		
	Speed	Distance computer mouse is moved divided by the duration of the movement
	Accuracy	Proportion of mouse events where the movement direction remained equal along the x-axis and y-axis
**Mouse events**		
	Clicks	Proportion of mouse tracks with clicks in a recording
	Wheels	Proportion of mouse tracks with wheels in a recording
**Recording time**		
	Weekday	Categorical {1: Monday, 2: Tuesday, 3: Wednesday, 4: Thursday, 5: Friday, 6: Saturday and Sunday}
	Daytime	Dummy with 1 if recording was in the morning, 0 otherwise (in the afternoon)
**Sociodemographics**		
	Age	Subject age
	Gender	Dummy with 1 if male, 0 otherwise (female)
	Education	Dummy with 1 if university degree, 0 otherwise (ie, high school or lower)
**Health and nutrition**		
	Sport	Hours of sport per week
	Nutrition	Number of fruits or vegetables consumed per day
	Alcohol	Categorical {1: never, 2: 2-4 times per month, 3: 2-3 times per week, 4: more than 4 times per week}
	Smoking	Categorical {1: daily, 2: occasionally, 3: not anymore, 4: never smoked}
Personality traits		The big five personality traits, each measured on a scale from 1 (low expression of the trait) to 10 (high expression of the trait), based on an established inventory [17]

### Processing Computer Mouse Movements

A Java application was developed to record computer mouse movements (ie, timestamp and x- and y-coordinates) and mouse events (ie, movement, click, and wheel). The application was built on the Windows operating system’s standard software drivers with a sample rate of approximately 125 Hz. Computer mouse movements were recorded for 30 minutes and processed in the following way. Each recording was split into separate trajectories, where a trajectory started with a mouse movement and ended with a different mouse event (ie, a click or wheel). Thereby, trajectories were only considered if their duration was between 1 and 10 seconds. This approach was beneficial, as it omitted trajectories that were extremely short or included temporary phases where the mouse was not moving. For each trajectory, two variables were computed: (1) mouse speed, which is the average movement speed, and (2) mouse accuracy, which is the proportion of mouse events where the direction of the movement remained equal along the x- and y-axes (ie, the proportion of times the movement direction was *not* corrected). Both variables were then averaged over all trajectories. These provided the features that were inserted into our regression model.

Mouse speed was computed as the total distance the mouse moved between the start time *t*=1 of a trajectory and its end time *T* divided by the trajectory’s total duration *T*. Hence, this yielded the following:



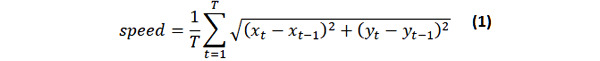



Mouse accuracy is the relative frequency of how often the movement in x- and y-directions was *not* changed. It is formalized by the following:



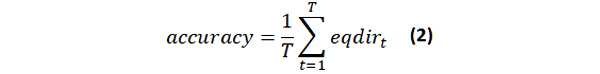



where the variable *eqdir_t_* indicates whether the movement in both x- and y-directions remained equal at time *t*. It returns a value of 1 if this is the case and 0 otherwise. Formally, it is specified by the following:



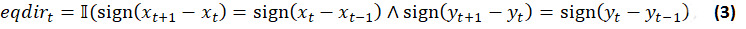



Accordingly, the larger the accuracy value is, the less the movement direction was altered. If the value for accuracy is 1, then the movement direction was never altered, and if the value for accuracy is 0, then the movement direction was always altered. In other words, the more accurate movement was the one with fewer corrections. This directly relates our measure of accuracy to the theory of neuromotor noise, which predicts more corrections as the movement speed is increased.

The proportion of direction changes is commonly used as a measure of accuracy in related work [18,19]. Another measure for accuracy is the deviation from an optimal trajectory [19,20]. However, the theoretical model underlying the speed-accuracy trade-off predicts that higher movement speed leads to more corrective submovements [13,14], not necessarily to a larger deviation from the optimal line between the start and end point of the trajectory. For that reason, we specifically chose the proportion of direction changes as our measure of accuracy in this study.

### Stress Measurement

Acute stress was measured according to the circumplex model of affect [21]. This model relates affective states to two underlying neurophysiological systems: valence, a pleasure-displeasure continuum, and arousal or alertness [22]. Both were collected using self-assessment manikins [23] on a 7-point Likert scale, with a value of 1 referring to a very negative valence (very low arousal) and a value of 7 indicating a very positive valence (very high arousal). Acute stress was then defined as a combination of low valence and high arousal, which has been shown to be related to work stressors in empirical research [24]. Specifically, stress is encoded as a dichotomous variable that equals 1 if subjects reported low valence and high arousal (ie, valence below the neutral midpoint of 4 and arousal above the neutral midpoint of 4) and 0 otherwise. Hence, our encoding translates into an analysis that focuses on distinguishing negative stress from positive or no stress.

### Statistical Analysis

A logistic regression model was estimated with stress as the dichotomous outcome variable and with features from computer mouse movements as the independent variables. The model is specified as follows:



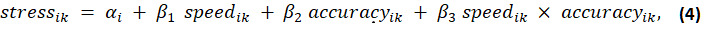



where *stress_ik_* is the dichotomous outcome variable for subject *i*=1,...,*M* and recording *k*=1,...,*N*. Subject-specific variation in average stress levels is captured by the varying intercept α*_i_*. Note that subject-specific characteristics such as age or gender could explain between-subject variation of average stress levels, but beyond that, time-varying variables such as computer mouse movements are needed to explain within-subject variation of stress levels over time. The association of mouse speed and accuracy with stress is estimated by β_1_ to β_3_. In particular, the two-way interaction between mouse speed and accuracy (β_3_) tests whether a speed-accuracy trade-off in computer mouse movements is associated with stress. Note that mouse speed and accuracy were centered and scaled by their empirical mean and standard deviation. By centering both variables, the sign of β_3_ indicates the direction of the speed-accuracy trade-off. That is, a negative sign of β_3_ would indicate that a simultaneous increase in mouse speed and decrease in mouse accuracy or a simultaneous decrease in mouse speed and increase in mouse accuracy is associated with a higher probability of stress.

Further independent variables were included in the above regression model as part of the sensitivity analysis. For instance, to control for mouse usage, we computed the number of events where the mouse was clicked or wheeled. Note that access to other human-computer interactions (eg, keyboard strokes) was not granted in this study due to privacy concerns.

### Model Estimation

A Bayesian approach was used for model estimation. Compared to classical statistics, the Bayesian approach requires the specification of priors for all model parameters. When choosing flat priors, the classical and Bayesian approaches are the same. However, when choosing a Bayesian prior (eg, a normal prior), the results are different, and sign errors are less frequent with a Bayesian prior [25]. In other words, a Bayesian approach is less prone to making wrong claims about the sign of a parameter. In our study setting, this would most likely give more conservative estimates; hence, a Bayesian approach was used for data analysis. We chose weakly informative priors for all model parameters, thereby following recommendations on the choice of priors [26]. Our priors are as follows:



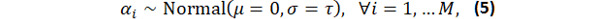





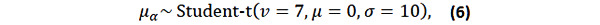





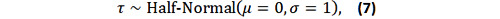





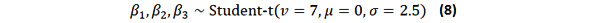



The model was estimated with Markov chain Monte Carlo using four chains. Each chain performed 2000 iterations divided into 1000 iterations for a warm-up and 1000 iterations for sampling. Samples were drawn with the No-U-Turn Sampler [27]. Thereby, it was ensured that all Markov chains converged successfully so that inference could be performed. In the Results section, we report the posterior distribution, the posterior mean, and the 95% highest posterior density interval (HPDI) of the estimated parameters.

Statistical analysis was performed with the programming language R, version 4.0.2 (The R Foundation), and the probabilistic programming language Stan, version 2.21.0 [28], using the interface provided by the R package brms, version 2.13.5 [29].

### Data Inclusion and Exclusion

All participants deciding to participate were included in the study (ie, no additional inclusion or exclusion criteria were applied). Our raw data contained 2029 recordings from 71 subjects. The number of recordings per subject varied due to absences or because the subjects decided to stop participating. Further, recordings were excluded when no computer mouse movements were recorded (5 recordings), the recorded computer mouse movements contained tracking errors (ie, incorrect time stamps) (92 recordings), or when the recordings contained less than 10 computer mouse trajectories (200 recordings). This led to the removal of 297 recordings from 62 subjects—between 1 and 12 per subject—and the exclusion of 1 subject from the study.

### Data and Code Availability

Preprocessed data and a script to replicate all model results are provided [30]. Raw data may be used to identify individual study participants (eg, because mouse movements can be very specific to a person or may reveal sensitive information, such as passwords, when using a software keyboard) and, thus, cannot be made available; this decision was made by the research team and the institutional review board that evaluated the study.

## Results

### Subject Statistics

Our results are based on 70 subjects and 1829 recordings (mean 26.13, SD 14.33). Subjects were between 20 and 61 years old, with a median age of 39.5 years (IQR 31.0-49.0). Further, 46% (32/70) of the participants were female, and 59% (41/70) held a university degree; all others had high school diplomas or lower. Recordings were roughly balanced by daytime hours (951/1829, 52.0% in the morning and 878/1829, 48.0% in the afternoon) and weekdays (329/1829, 18.0% to 384/1829, 21.0% per weekday and 18/1829, 1% on the weekend).

**Figure 1:**
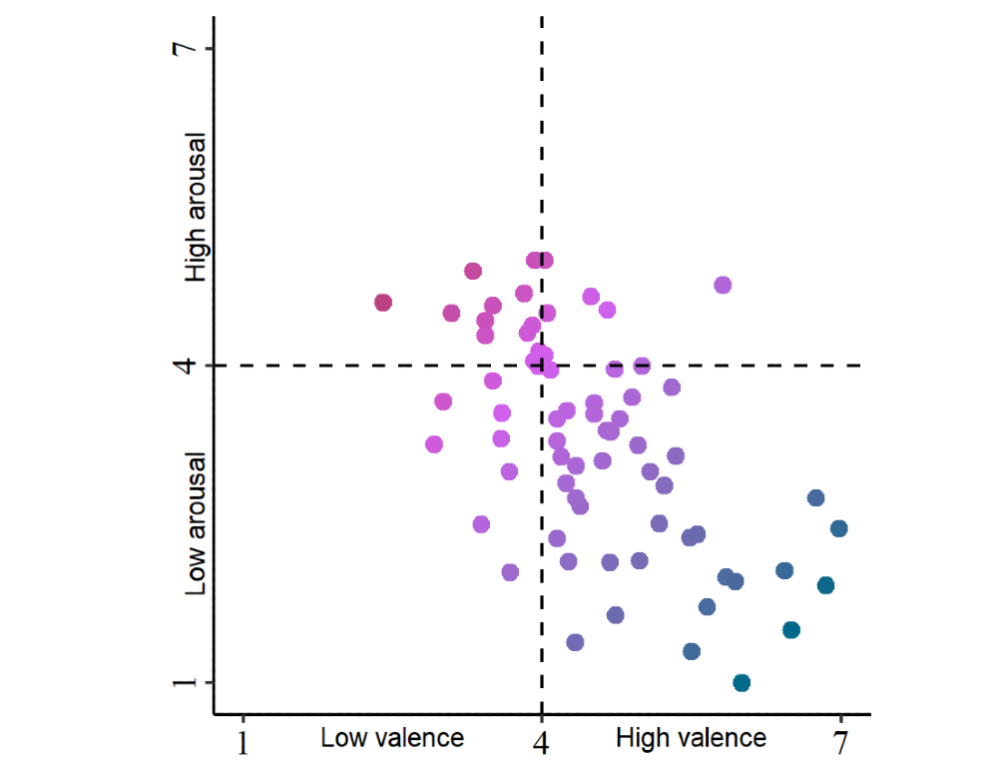
Perceived valence and arousal by subject. Shown are the average self-reported valence and arousal levels by subject in the field study. Red points indicate high levels and blue points indicate low levels of average stress.

Both valence and arousal varied across subjects (see Figure 1). Average valence per subject was slightly above the neutral midpoint (mean 4.53, SD 0.98), and average arousal was slightly below the neutral midpoint (mean 3.28, SD 1.02). When averaged over the study period, a combination of low valence and high arousal (the top-left quadrant in Figure 1) was observed in 12 out of the 70 (17%) subjects. Applying our encoding of stress following the circumplex model of affect [21], 185 out of the 1829 self-reports (10.1%) were classified as stressful.

### Association Between Stress and Computer Mouse Movements

**Figure 2:**
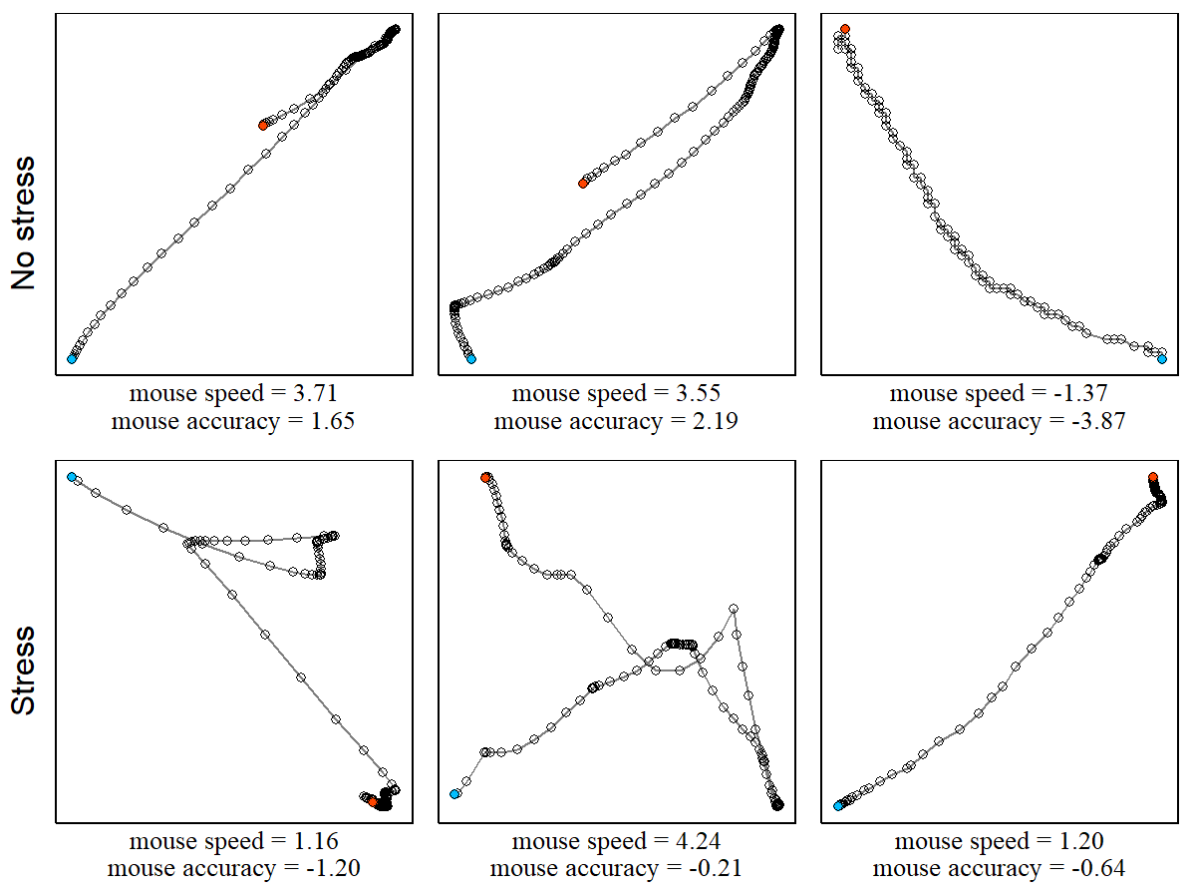
Illustrative examples of the speed-accuracy trade-off in computer mouse movements. Shown are typical computer mouse movements (blue dot: beginning of movement; red dot: click) from the field study. Circles correspond to recordings at 125 Hz. When subjects perceived no stress, computer mouse movements were typically not characterized by a speed-accuracy trade-off. When subjects perceived stress, computer mouse movements were typically characterized by a speed-accuracy trade-off. Mouse speed and accuracy were standardized to indicate the direction of the trade-off; that is, high speed (+) and low accuracy (−) or low speed (−) and high accuracy (+).

It was hypothesized that stress is characterized by a speed-accuracy trade-off. This trade-off is illustrated in Figure 2. When subjects perceived no stress, computer mouse movements were typically not characterized by a speed-accuracy trade-off. In contrast to that, when subjects perceive stress, computer mouse movements were typically characterized by a trade-off where the mouse was moved quickly but less accurately (ie, many direction changes) or slowly but more accurately (ie, few direction changes). Descriptives for average mouse speed and accuracy are provided in Multimedia Appendix 1, Figure S1.

**Figure 3:**
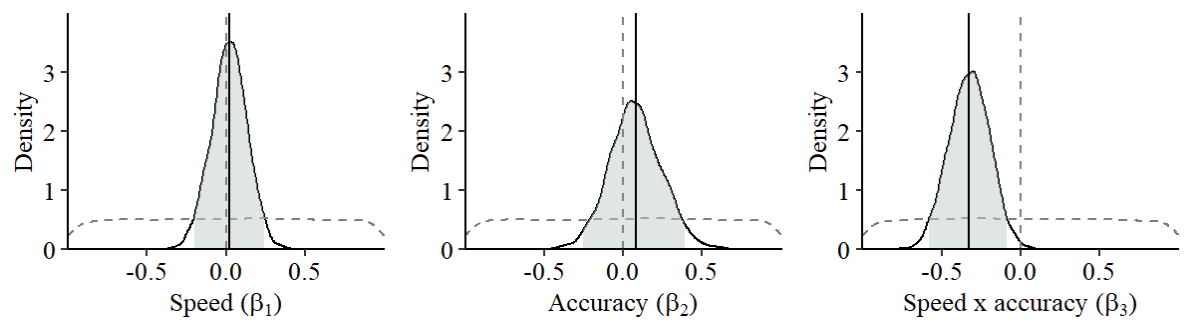
Association between work stress and computer mouse movements. Shown is the estimated effect (posterior and prior density and mean as solid and dashed grey lines, respectively, and 95% highest posterior density interval as shaded area) of mouse speed (β_1_), mouse accuracy (β_2_), and the two-way interaction between mouse speed and accuracy (β_2_).

The estimated parameters of mouse speed and accuracy were as follows. The individual parameters of mouse speed (β_1_) and accuracy (β_2_) were not significant based on the observation that the 95% HPDIs include zero (see Figure 3). However, the parameter for the two-way interaction between speed and accuracy (β_3_) was significant (mean −0.32, 95% HPDI −0.58 to −0.08). On average, a simultaneous 1 SD increase in mouse speed and 1 SD decrease in mouse accuracy, or vice versa, was associated with a change in the odds for perceiving stress by 1.53. In other words, work stress was characterized by a speed-accuracy trade-off.

**Figure 4:**
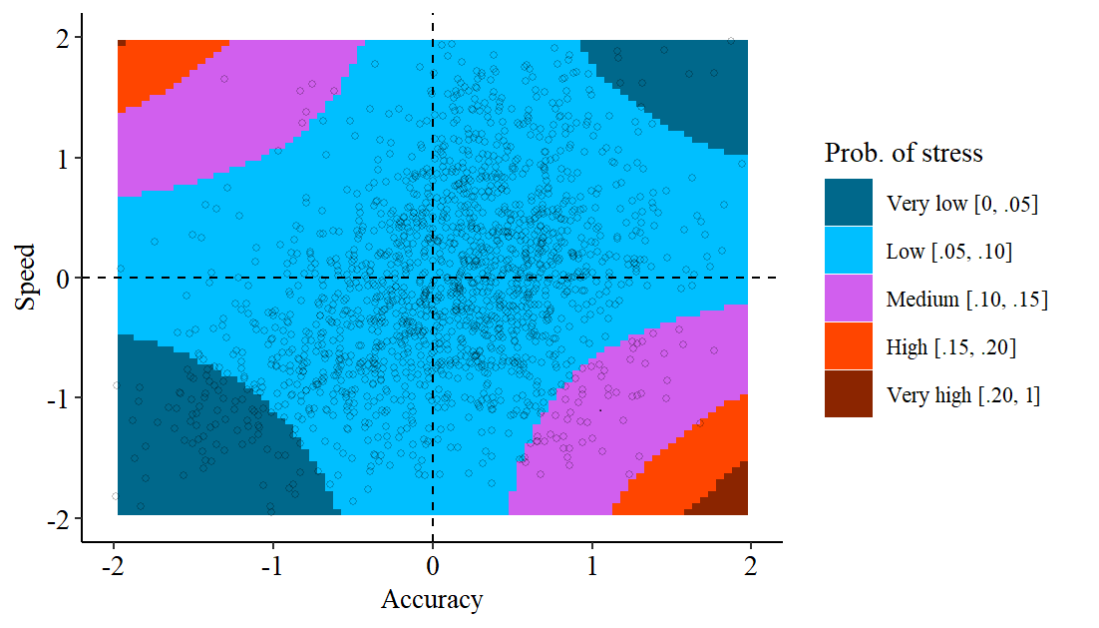
Probability (Prob.) of perceived stress based on mouse speed and accuracy. Shown is the partial dependence of stress on mouse speed and accuracy in the range of −2 SD to +2 SD. Red areas indicate high levels and blue areas indicate low levels of stress.

Figure 4 depicts the partial dependence of both mouse speed and mouse accuracy on the probability of perceiving stress. Based on the plot, two findings can be derived. First, stress was more likely when there was a speed-accuracy trade-off. Second, this trade-off seemed slightly more prevalent for low mouse speed and high mouse accuracy, as indicated by a higher share of observations in the lower-right corner. This means that although both directions of the trade-off are present in our data, subjects perceiving stress were slightly more frequently increasing accuracy at the cost of speed.

### Sensitivity Analysis

The sensitivity of the estimated parameters was assessed in the following ways. First, different processing of the data led to conclusive findings. In the above analysis, recordings were removed when fewer than 10 computer mouse trajectories were counted over 30 minutes. When varying this number, the estimated parameter of the mouse speed-accuracy trade-off remained stable (see Multimedia Appendix 1, Figure S2). Similarly, the maximum duration for a trajectory was set to 10 seconds. When varying the maximum duration from 5 to 20 seconds, the estimated parameter of the mouse speed-accuracy trade-off also remained stable (see Multimedia Appendix 1, Figure S3). Furthermore, recordings from 2 subjects revealed unusually low mouse accuracy (see Multimedia Appendix 1, Figure S1). Excluding all recordings from these subjects slightly reduced the size of the estimated parameter for the trade-off (mean −0.22, 95% HPDI −0.42 to −0.03).

Second, the sensitivity of the estimated parameter for the speed-accuracy trade-off was assessed with respect to the inclusion of varying slopes for computer mouse movement variables and additional controls, such as mouse events and sociodemographics. Including varying slopes or adding more controls led to comparable estimates for the parameter of the mouse speed-accuracy trade-off (see Multimedia Appendix 1, Figure S4).

Third, the association of computer mouse movements with valence, arousal, and a discrete measure of stress (defined as arousal – valence + 6) was estimated. Results from Poisson regressions with the same model specification showed no significant associations but a tendency that arousal and the discrete measure of stress were negatively associated with the speed-accuracy trade-off (see Multimedia Appendix 1, Figure S5). However, the results from the regression with the discrete measure of stress as the outcome should be interpreted with caution, as an arousal level of 7 and valence level of 4 would result in the equivalent level of stress as an arousal level of 5 and a valence level of 2, whereas only the second self-report would be labeled as stress according to the circumplex model of affect.

Fourth, the possibility of selection bias was investigated, with a statistical comparison between those subjects with few (n≤10) and many (n>10) recordings. The proportion of recordings with stress from subjects with few recordings (6/43, 14%) was higher than the proportion of recordings with stress from subjects with many recordings (188/1986, 9.5%). However, the difference was not statistically significant (χ^2^_1_=0.5, *P*=.47). This result suggests that participation intensity was not significantly related to stress. Other than that, it could not be investigated whether individuals outside this study were more or less stressed than the subjects participating in this study.

## Discussion

### Principal Findings

The goal of this study was to examine whether computer mouse movements indicate work stress. Data from a 7-week longitudinal field study supported the hypothesis. Despite the heterogeneity of computer tasks and the resulting complexity of computer mouse movements, we found a significant association with work stress. That is, work stress was characterized by a speed-accuracy trade-off in computer mouse movements.

### Comparison With Prior Work

This is the first study to infer stress from the computer mouse in the field (ie, at the workplace). In prior work, lab studies were conducted to investigate the association between stress and the use of the computer mouse [7-11]. In these lab studies, subjects performed artificial tasks (eg, point-and-click tasks) in a controlled environment. In contrast to that, our data were collected unobtrusively while subjects were performing office work in a real-world environment. On the one hand, this made data processing and analysis challenging. On the other hand, it provided us with the unique opportunity to present the first empirical evidence as to whether stress is associated with computer mouse movements in the field.

A drawback of our field study in comparison to the lab studies is that we are not able to estimate a causal link. The reason is that there are potentially unmeasured confounders. In particular, computer mouse movements as well as stress may depend on the difficulty of the task, with more difficult tasks resulting in higher levels of stress. In the lab, it is possible to control which task is performed, whereas this is not possible in the field without obtrusive monitoring of tasks. However, precisely because unobtrusive and continuous monitoring of tasks is not feasible in the field, computer mouse movements may be a good proxy for how stressful a task is perceived and may thus provide an indirect way to measure stress.

### Benefits

Monitoring of computer mouse movements provide a number of benefits for stress management in the workplace. Most office work involves computer tasks; as such, computer mouse movement data are readily available. Unlike other forms of stress monitoring, computer mouse movements present a viable tool for monitoring stress at scale because they can be collected in an unobtrusive fashion and continuously over time [10]. The latter becomes important when offering on-demand stress management interventions by organizations and for monitoring their effectiveness [31]. It is also possible to monitor stress by monitoring physiological changes (eg, heart rate variability or skin conductivity) through wearable devices. However, when introduced by employers, the broad usage of physiological data in a corporate context raises issues regarding their acceptance and legitimacy [32]. When compared to such physiological stress measurements, many employees might consider the measurement of computer mouse movements as a clearly work-related behavior and as a less intrusive and more legitimate monitoring method at work. As computer mouse movements are bound to currently performed work, their measurement will trigger a more balanced action on the part of employees to mitigate work stress: both reducing their own receptivity to stress and improving the underlying working conditions, as it is also recommended by the European Union [33]. Thus, the measurement of computer mouse movements offers a valuable, complementary approach to physiological measurements.

### Limitations

Our study also has limitations. First, our work constitutes an observational study with an explanatory analysis of the data. As a consequence, a causal interpretation of the estimated association is precluded. Second, computer mouse movements were only linked to the presence of stress, which was defined according to the circumplex model of affect [21]. The severity of stress could not be assessed due to the low prevalence of high levels of stress. Third, computer mouse movements were only linked to acute stress. The association of computer mouse movements with chronic stress is subject to future work. Fourth, the outcome of this study was psychological stress, which was measured based on self-reports. It is unclear if, and to what extent, psychological and physiological measures of stress are alternative or complementary by nature [34]. Thus, collecting physiological data from wearable devices to monitor stress [35,36] could be used to validate the association with computer mouse movements. Fifth, the sources of stress were not identified, which is important for managing stress. However, other work suggests that human-computer interactions also correlate with workplace stressors [37]. Sixth, the determinants for the directions of the speed-accuracy trade-off were not explored. This would probably require a different research setting, most likely a controlled lab experiment, in order to investigate what causes subjects to increase speed at the cost of accuracy, or vice versa.

### Conclusions

To summarize, the findings of this study suggest that the computer mouse can be used to infer work stress. These findings could be combined with findings from other forms of human-computer interactions (eg, computer trackpads [38] or keyboard strokes [39]) in order to develop digital tools for detecting stress.

## Appendix

Multimedia Appendix 1 Supplementary figures for descriptives and sensitivity analyses.

## Abbreviations

HPDI: highest posterior density interval
