# Supplementary material for: Computer Mouse Movements as an Indicator of Work Stress: Longitudinal Observational Field Study
Source: J Med Internet Res. 2021 Apr 2;23(4):e27121. doi: 10.2196/27121 (PMC8052599; doi:10.2196/27121)
Supplement: Multimedia Appendix 1 [file jmir_v23i4e27121_app1.docx]

# Multimedia Appendix 1. Supplementary figures for descriptives and sensitivity analyses.

## Descriptives


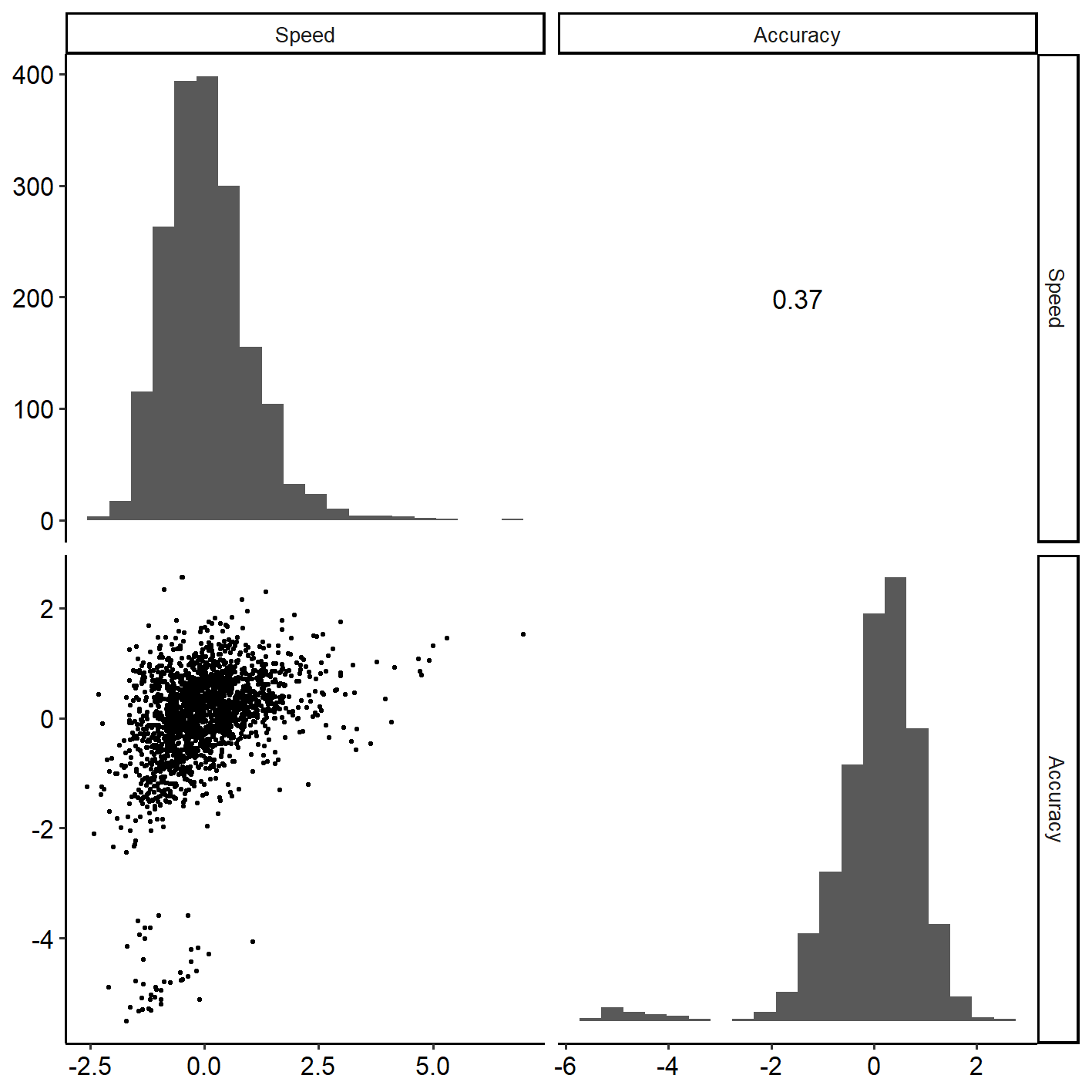


**Figure S1**: Mouse speed and accuracy after data preprocessing. The frequency of average mouse speed and accuracy are shown on the diagonal, and the bivariate correlation between speed and accuracy is shown on the anti-diagonal (r = 0.37).

## Sensitivity analysis


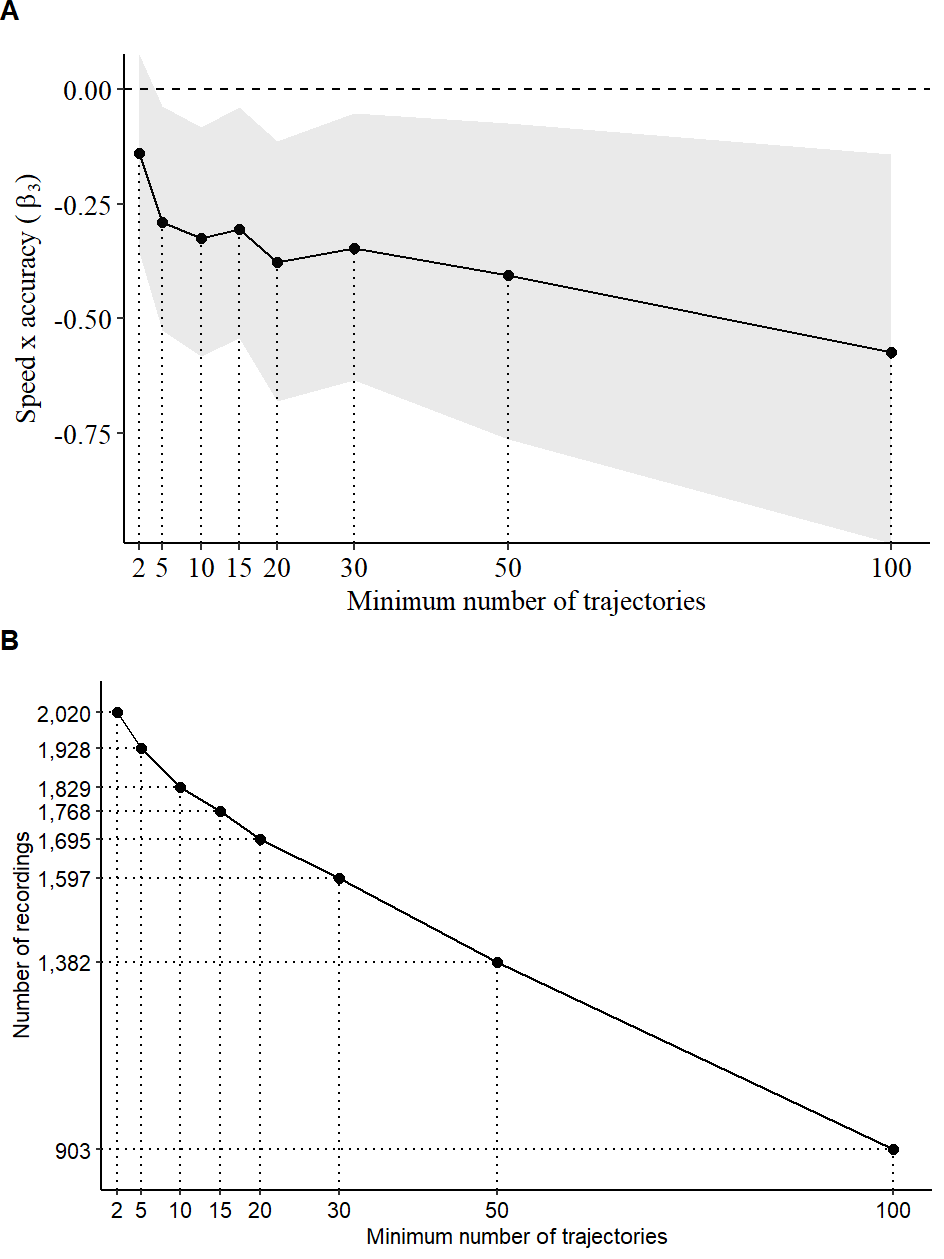


**Figure S2**: Sensitivity of the speed-accuracy tradeoff when varying the minimum number of trajectories. **A** Estimated parameter (posterior mean and 95% HPDI) of the interaction between speed and accuracy ($\beta_{3}$) when varying the minimum number of trajectories set to compute mouse speed and accuracy. **B** The number of recording corresponding to the minimum number of trajectories. Note that more and more recordings are removed from the model as the minimum number of trajectories is increased.


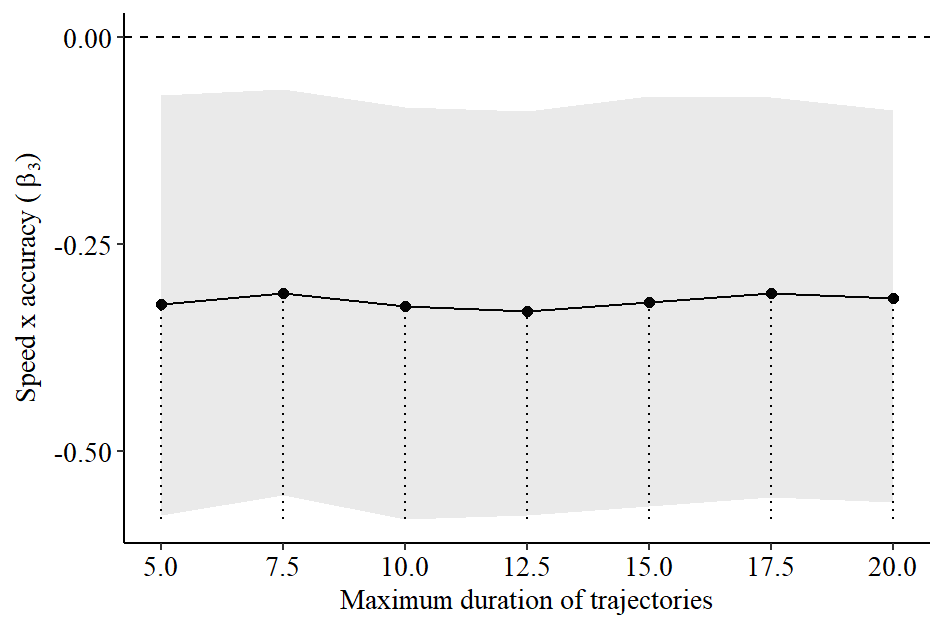


**Figure S3**: Sensitivity of the speed-accuracy tradeoff when varying the maximum duration of trajectories. Shown is the estimated parameter (posterior mean and 95% HPDI) of the interaction between speed and accuracy ($\beta_{3}$) when varying the minimum number of trajectories set to compute mouse speed and accuracy.


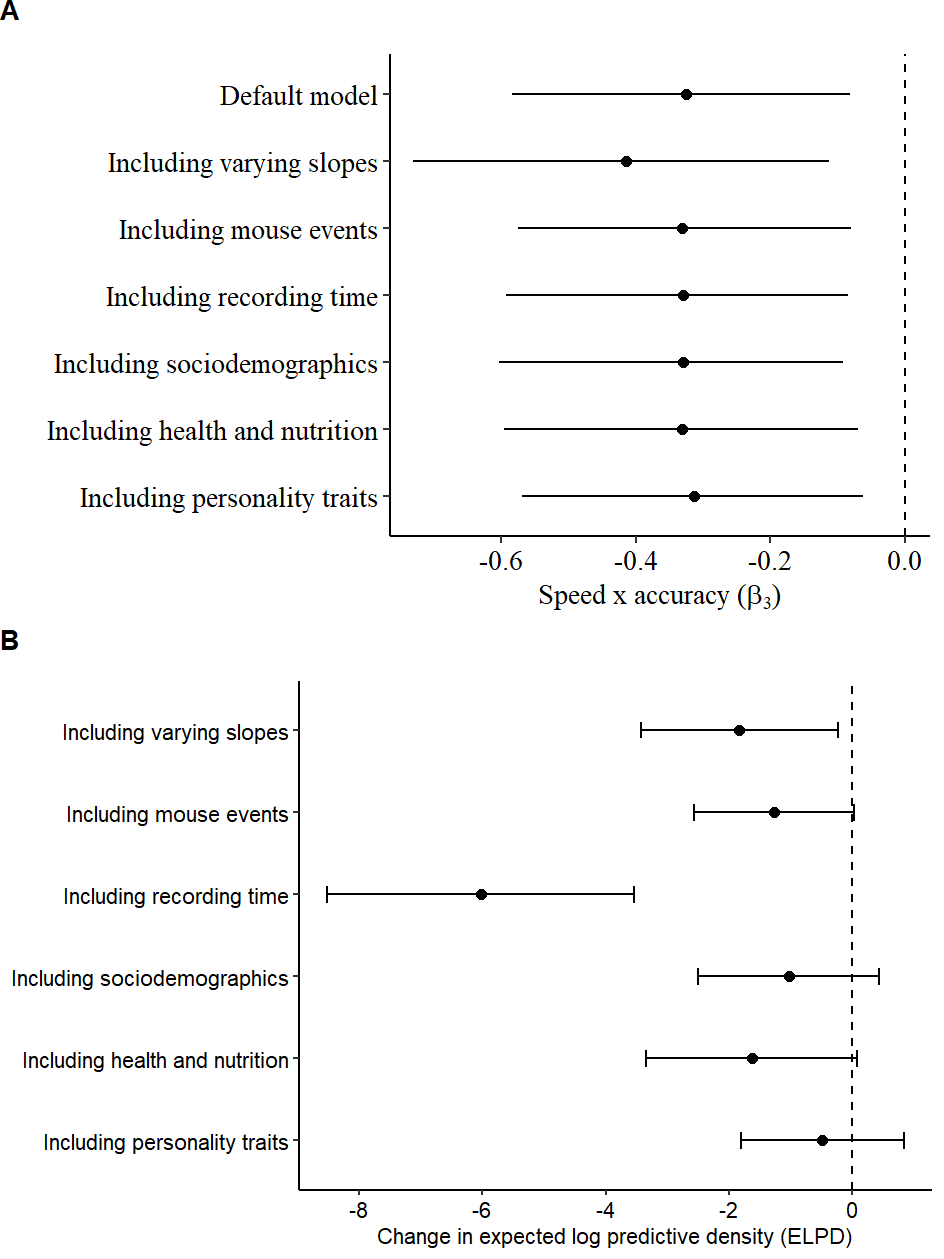


**Figure S4:** Sensitivity of the speed-accuracy tradeoff when including varying slopes and additional control variables. **A** Estimated parameter (posterior mean and 95% HPDI) of the interaction between mouse speed and accuracy $(\beta_{3}$). **B** Change in model information criteria over the default model as measured by the change in expected log predictive density (ELPD±SE), which is estimated from Pareto smoothed importance sampling leave-one-out cross-validation (PSIS-LOO).


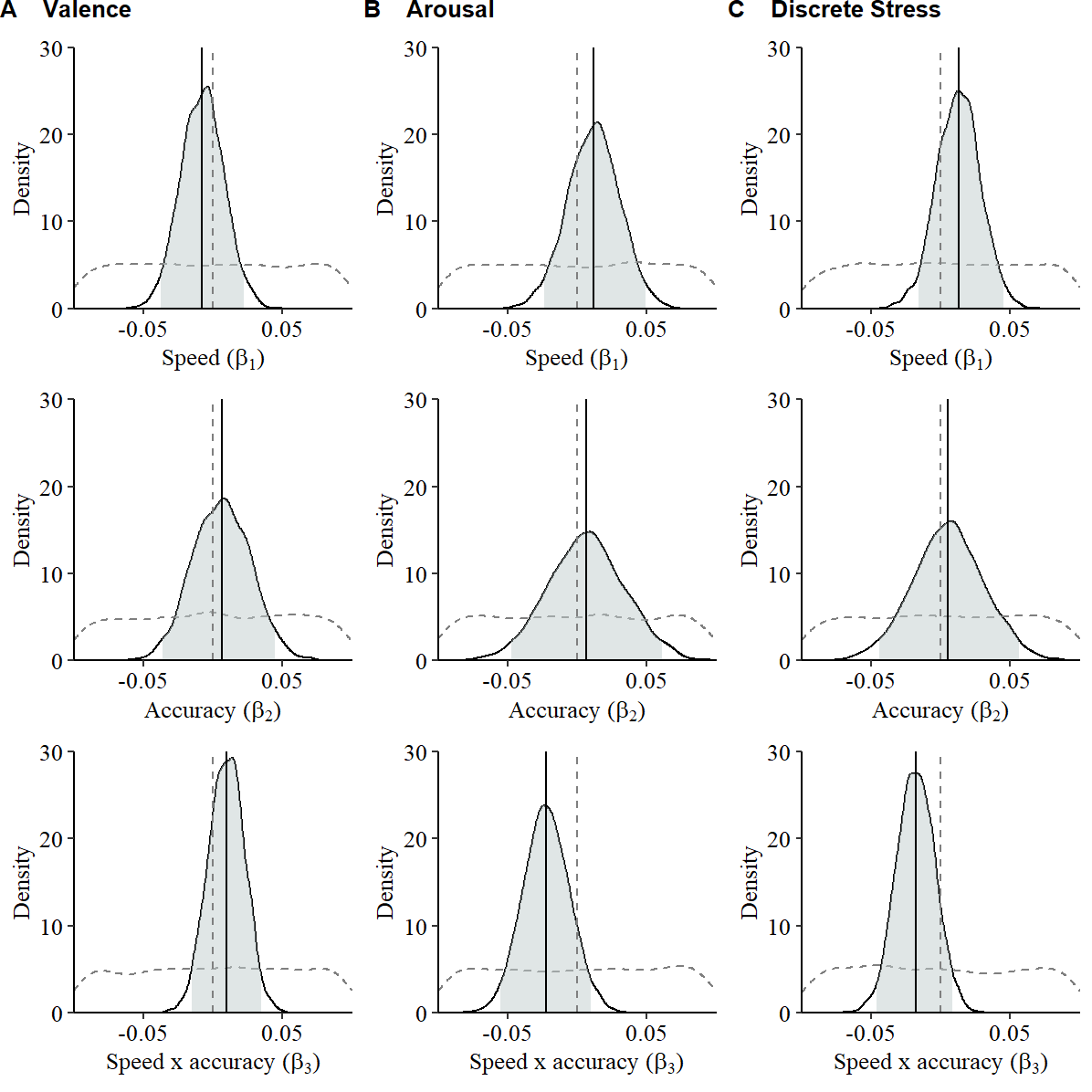


**Figure S5:** The association of CMMs with valence, arousal, and a discrete measure of stress (defined as valence – arousal + 6). **A-C** Estimated parameters (posterior mean and 95% HPDI) of mouse speed $(\beta_{1}$), mouse accuracy $(\beta_{2}$)., and the interaction between mouse speed and accuracy $(\beta_{3}$) when the outcome is valence (**A**), arousal (**B**), or discrete stress (**C**), respectively. A Poisson regression was estimated for each outcome with the same model specification that was used for estimating the association with the dichotomous stress outcome.
